# Early psychosocial-behavioral phenotypes associated with subsequent engagement and longitudinal outcomes in a digital health intervention for heart failure

**DOI:** 10.1093/abm/kaag066

**Published:** 2026-11-02

**Authors:** Namuun Clifford, Rachel Tunis, Qingyi Zeng, Shelli R Kesler, Kavita Radhakrishnan

**Affiliations:** National Clinician Scholars Program, University of California, San Francisco, San Francisco, CA 94158, United States; School of Nursing, The University of Texas at Austin, Austin, TX 78701, United States; School of Nursing, The University of Texas at Austin, Austin, TX 78701, United States; School of Nursing, The University of Texas at Austin, Austin, TX 78701, United States; School of Nursing, The University of Texas at Austin, Austin, TX 78701, United States; School of Nursing, The University of Texas at Austin, Austin, TX 78701, United States

**Keywords:** heart failure, digital health interventions, phenotypes, patient engagement, ecological momentary assessment, self-care behavior

## Abstract

**Background:**

The effectiveness of digital interventions for heart failure (HF) is limited by variable engagement. Psychosocial phenotyping can characterize engagement heterogeneity, but it remains unclear whether early phenotypes are associated with subsequent engagement and HF outcomes.

**Purpose:**

To identify early psychosocial-behavioral phenotypes and examine associations with longitudinal affective and motivational states, digital engagement, and HF outcomes.

**Methods:**

We conducted a secondary analysis of a 6-month decentralized randomized controlled trial, using *k*-medoids clustering of baseline psychosocial characteristics and the first 4 weeks of device engagement among 175 adults with HF. Kruskal-Wallis tests examined phenotype differences in engagement during weeks 5-24. Linear mixed-effects models tested differences in anxiety, depressed mood, motivation, and patient-reported outcomes across 24 weeks.

**Results:**

Three phenotypes emerged: Engaged Self-Regulators (42%), Activated Learners (31%), and Challenged Survivors (26%), characterized by psychological profiles, social and structural resources, and early engagement patterns. Phenotypes differed in subsequent device engagement (all *P* values less than or equal to .03), with Engaged maintaining the highest use. Differences in anxiety, depressed mood, and motivation persisted over time (*P* < .001). All phenotypes improved in functional status, quality of life, and self-care; however, Engaged maintained functional status and quality of life scores 20-23 points higher than both other phenotypes (*P* < .001). Heart failure knowledge trajectories diverged; Engaged and Challenged improved comparably, while Activated showed no gains (*P* = .008).

**Conclusions:**

Psychosocial-behavioral phenotypes identified early in a digital intervention were associated with engagement and HF outcomes. Early phenotyping may provide a foundation for prospectively testing phenotype-informed tailoring of digital interventions.

**Parent Trial Registration:**

ClinicalTrials.gov: NCT05056129.

## Introduction

Heart failure (HF) is a chronic, progressive condition affecting 6.7 million adults in the United States, with 1 in 4 expected to develop it in their lifetime.^1^ Despite decades of advances in treatment, HF remains the leading cause of all cardiovascular hospitalizations and nearly half (45%) of all cardiovascular deaths.^1^^,^^2^ Characterized by high symptom burden, psychological distress, and poor quality of life (QoL), HF imposes considerable costs on patients, caregivers, and health systems.^1^^,^^3^ Although clinical care is essential for disease management, most HF management occurs outside the health-care system, making self-care—defined as the process of maintaining health, monitoring symptoms, and managing illness—the cornerstone of effective HF management.^4–6^ Heart failure self-care behaviors are associated with improved QoL and reduced hospitalizations and mortality.^4^^,^^5^^,^^7^^,^^8^ However, fewer than 10% of patients adhere to all recommended behaviors, with particularly low adherence for physical activity, diet, and daily weight monitoring.^9^

Digital health interventions (DHIs) employing wearable devices, telemonitoring, and mobile apps offer a promising approach to support HF self-care by facilitating daily symptom and weight monitoring, providing tailored education and feedback, and empowering patients to become active agents in their own care.^10^ Growing evidence indicates that DHIs improve self-care knowledge and behaviors and reduce hospitalizations and mortality.^11–13^ However, the effectiveness of these interventions depends largely on some degree of engagement with the digital tools.^14^^,^^15^

Two decades ago, Eysenbach^16^ introduced the “law of attrition,” describing a common phenomenon in digital health trials in which large proportions of participants disengage or drop out early. Since then, despite advancements in technology, intervention design, and behavioral science, high attrition and inconsistent user engagement continue to undermine the promise of DHIs.^17^ Systematic reviews of DHI engagement in HF have found substantial variability, with adherence ranging from 31% to 98% across studies, and consistent declines in use over time.^10^^,^^18^ Lower engagement has been observed among individuals of lower socioeconomic status, racial and ethnic minorities, and those with limited technology experience.^15^^,^^19–22^ Differential engagement with DHIs may contribute to intervention-generated inequalities, disproportionately benefiting more advantaged groups and introducing biases that limit generalizability and may exacerbate existing health disparities.^22^^,^^23^

Despite the importance of engagement to DHI success, critical gaps remain in understanding why engagement varies across individuals and whether distinct engagement profiles are associated with intervention outcomes.^16^^,^^24^^,^^25^ Prior work has often operationalized engagement solely through behavioral usage metrics, overlooking the affective, cognitive, and contextual elements that shape meaningful engagement.^10^^,^^15^^,^^24^^,^^25^ Psychosocial-behavioral phenotyping offer a data-driven approach to capturing this heterogeneity by identifying subgroups of individuals who share distinct patterns across psychological, social, and behavioral characteristics.^26^^,^^27^ Such multidimensional profiles may help identify individuals with different engagement patterns and intervention responses. Furthermore, associations between these profiles and longitudinal outcomes are underexplored.^25^^,^^28^

To move beyond descriptive usage metrics and understand why engagement varies and how it shapes outcomes, we draw upon the AIM-ACT framework.^24^ The framework posits that an individual’s *affective*, *integrative*, and *motivational* responses—shaped by *attention* and *context*—drive engagement with digital interventions and support the *translation* of motivation into behavior.^24^ In prior work guided by the AIM-ACT framework, we empirically derived psychosocial phenotypes to characterize digital engagement among 146 adults with HF using a mixed-methods design.^29^ Three subgroups emerged—Challenged Survivors, Activated Learners, Engaged Self-Regulators—characterized by meaningful differences in affective-motivational profiles, social resources, and device engagement patterns.^29^ Although these phenotypes provided a multidimensional characterization of engagement heterogeneity, several questions remained. Because phenotypes were derived using device engagement data across the full 6-month intervention, they could only be identified retrospectively. It remains unknown whether comparable subgroups can be identified using only early engagement data and whether phenotype membership is associated with subsequent digital engagement and longitudinal patient-reported outcomes. It is also unclear whether the affective-motivational states assessed via ecological momentary assessments (EMA) remain differentiated over time or reflect transient experiences. Addressing these questions is necessary to evaluate the potential utility of early psychosocial-behavioral phenotyping for future DHI tailoring.

We address these gaps in a secondary analysis of 175 adults with HF from a 6-month, fully decentralized randomized controlled trial. We aimed to: (1) derive psychosocial-behavioral phenotypes using baseline psychosocial characteristics and the first 4 weeks of device engagement; (2) characterize baseline differences across phenotypes and their associations with subsequent device engagement during weeks 5-24; (3) evaluate whether phenotype-defining affective-motivational states remained differentiated over time using longitudinal EMA data; and (4) examine whether phenotype membership was associated with patient-reported outcomes for HF functional status, QoL, self-care behaviors, and knowledge across 24 weeks. By linking early psychosocial-behavioral phenotypes to subsequent digital engagement and longitudinal patient-reported outcomes, this study extends prior phenotyping work and may inform future development of phenotype-tailored DHIs for adults with HF.

## Methods

### Study design and sample

We conducted a secondary analysis of data collected between 2022 and 2025 from a 6-month decentralized randomized controlled trial.^30^ The methods have been described elsewhere,^30^ but briefly, the parent trial enrolled adults with HF from 20 US states with high HF burden. Eligible participants were English-speaking adults aged ≥45 years with NYHA class I-III HF and a documented HF-related hospitalization within the past 12 months.

All participants were provided digital tools including a smart scale, activity tracker, and a mobile app for data tracking. Participants were randomized 1:1 (stratified by sex and age) to an attention control group or an intervention group that additionally received a digital game app designed to motivate adherence to self-care behaviors.^31^ Surveys were completed online through REDCap, and behavioral data from smart scales and activity trackers were collected for 168 consecutive days from device logs. We combined both arms for all analyses because both groups received the same sensor devices and included group assignment as a covariate in outcome models to adjust for any intervention effects.

Of 200 enrolled participants, 25 were excluded from this analysis (16 never installed study devices; 9 lacked baseline EMA data required for clustering), resulting in a final sample of 175 participants. We followed the Strengthening the Reporting of Observational Studies in Epidemiology (STROBE) guidelines for reporting.^32^ The study was approved by the Institutional Review Board of The University of Texas at Austin, and all participants provided informed consent.

### Measures

#### Baseline characteristics

At enrollment, participants completed online baseline surveys reporting age (years), sex (male/female), race and ethnicity (categorized as non-Hispanic White vs racial/ethnic minority), HF duration (≤1 year, 2-4 years, ≥5 years), employment status (employed, retired, disabled, unemployed), smartphone type (Android/iOS), and prior fitness tracker ownership (yes/no). The Upstream Risk Screening Tool was used to assess social determinants of health, including food insecurity, housing concern, transportation difficulty, and perceived neighborhood safety (all yes/no). A full list of study variables, data sources, and scoring is included in Table S1.

#### Clustering variables

We used 10 variables from psychological, social, and behavioral domains for clustering, consistent with our prior study.^29^ For psychological variables, we used week 1 mean scores of daily morning EMAs completed over 7 consecutive days to capture baseline levels of momentary anxiety, depressed mood, and physical activity motivation, measured from 0 to 100 on a visual analog scale. Self-efficacy for HF self-care was measured at baseline using the SCHFI-6 Self-Care Confidence subscale (0-100; α = .83).^33^ Social variables included social connectedness (4-item composite, range 0-8), education (≤high school vs college or higher), financial hardship (yes/no), and partnered status (yes/no). Digital engagement was operationalized as the number of days each device (activity tracker, smart scale) was synced during the first 4 weeks of the study (days 1-28), entered as separate variables. The 4-week window was selected based on evidence that engagement within the first few weeks of digital interventions predicts subsequent use, while ensuring all clustering inputs preceded both the subsequent engagement period (weeks 5-24) and patient-reported outcome assessments at 6, 12, and 24 weeks.^34^

#### EMA outcomes

Anxiety, depressed mood, and physical activity motivation were measured via daily EMAs for 7 consecutive days at 4 timepoints (1, 7, 13, 23 weeks). Person-level means were calculated by averaging daily morning EMA responses within each wave. Week 1 mean scores were used as clustering inputs and means across all 4 waves were examined to assess whether between-phenotype differences in phenotype-defining affective-motivational states persisted over time.

#### Patient-reported HF outcomes

Four HF-related patient-reported outcomes were measured using self-reported surveys at 4 timepoints (baseline and weeks 6, 12, and 24). All measures were standardized to 0-100 with higher scores indicating better status. Functional status was measured using the Kansas City Cardiomyopathy Questionnaire combining the physical limitation and total symptom domains (KCCQ-FS; α = .93).^35^ Quality of life was assessed using the KCCQ-QoL subscale (KCCQ-QoL; α = .78).^35^ Self-care behaviors were measured using the European Heart Failure Self-care Behavior Scale (EHFScBS-9; α = .80).^36^ Heart failure knowledge was assessed using the Atlanta HF Knowledge Test (AHFKT; α = .87).^37^

### Analytic approach

#### Phenotype derivation and profiling

We conducted *k*-medoids clustering, also known as Partitioning Around Medoids (PAM) with Gower distance to identify psychosocial-behavioral phenotypes. The PAM algorithm was chosen because of its robustness to outliers and compatibility with mixed data types.^38^ We ran the algorithm for *k* = 2-10 and selected the final solution based on the elbow plot, average silhouette width, bootstrap stability (Jaccard index, 500 resamples), and interpretability.^39^^,^^40^ We also cross-tabulated assignments between competing cluster solutions to examine how they differed. Continuous variables were *z*-score normalized prior to clustering; categorical variables were treated as factors.

As a sensitivity analysis, we repeated the clustering using device engagement over the full 6-month study period consistent with our prior work,^29^ and assessed agreement with the 4-week solution using percent concordance and the adjusted Rand index. To compare phenotypes on baseline characteristics and subsequent device engagement (weeks 5-24), we used Kruskal-Wallis tests for continuous variables and chi-square tests for categorical variables. For significant Kruskal-Wallis tests, we used Dunn’s post hoc tests with Benjamini-Hochberg correction for pairwise comparisons.^41^

#### Linear mixed-effects models

We fit 2 sets of LMMs, with final models estimated using restricted maximum likelihood and the Engaged phenotype as the reference group. Fixed effects were evaluated using Type III *F*-tests with Satterthwaite degrees of freedom. Estimated marginal means and pairwise comparisons used Tukey adjustment. To control for false discovery rates across related models, we applied Benjamini-Hochberg correction to omnibus phenotype tests across the 3 EMA models and separately across the 4 health outcome models. For EMA affective-motivational states, random-intercept LMMs included phenotype, categorical study wave, and their interaction without covariates, to assess whether phenotype-defining differences observed at week 1 remained differentiated across subsequent EMA waves. Complete-case sensitivity analyses were conducted for the EMA models (Supplementary Material S1).

For patient-reported outcomes (functional status, QoL, self-care behaviors, HF knowledge), time was coded in weeks (0, 6, 12, 24), centering the intercept at baseline. We evaluated random slopes for time and retained them when they significantly improved model fit. Models were built sequentially: (1) unconditional growth; (2) addition of phenotype; (3) addition of covariates (age [centered], sex, minority status, study group assignment, and prior tracker ownership); and (4) addition of a phenotype × time interaction. We selected covariates a priori based on their associations with HF outcomes and digital engagement.^19–22^ Nested models were compared using likelihood ratio tests, retaining interaction terms only when they significantly improved fit. We conducted sensitivity analyses by excluding observations with standardized residuals exceeding |3|, which produced substantially similar results. Model diagnostics indicated no meaningful violations, and multicollinearity was minimal (all variance inflation factors < 2).

#### Missing data

Missing data increased over time and differed by phenotype for both EMA measures and patient-reported HF outcomes (Tables S2 and S3). Overall, 14% of person-wave EMA observations were missing, with the highest missingness in the Challenged phenotype (35% missing by week 23, vs 18% for Engaged). For HF outcomes, missingness followed a predominantly monotone dropout pattern; by week 24, rates ranged from 22% to 28% in Challenged and 26% in Activated participants, compared with 11%-12% in Engaged. For the HF outcome models, logistic regression indicated that younger age and intervention assignment predicted missingness; both variables were included as covariates in the outcome models. Because we could not rule out that data were missing not at random, we also conducted pattern-mixture models and tipping point analyses. Full missing data procedures and results are reported in Supplementary Material S1. All analyses were conducted in the R Statistical Package version 4.3.2 using the *cluster, fpc, lme4, lmerTest*, and *emmeans* packages.^42^ Statistical tests were 2-sided with a significance level of α = .05.

## Results

### Psychosocial-behavioral phenotypes

Among 175 participants, the mean age was 59.5 years (SD = 9.3; range 45-89), and 108 (62%) were male. Participants identified as White (*n* = 143, 82%), African American (*n* = 23, 13%), American Indian/Alaska Native (*n* = 3, 1.7%), Asian (*n* = 1, 0.6%), or another race (*n* = 5, 2.9%); 11 (6%) identified as Hispanic. For analysis, race and ethnicity were combined into non-Hispanic White (*n* = 135, 77%) vs racial or ethnic minority (*n* = 40, 23%). Baseline participant characteristics are reported in Table 1.

**Table 1:** Baseline characteristics by psychosocial-behavioral phenotype (*N* = 175).

Variable	Challenged Survivors (*n* = 46)	Activated Learners (*n* = 55)	Engaged Self-Regulators (*n* = 74)	*H*/χ^2^	*P*
**Demographics**					
** Age (years), *M* (SD)**	55.6 (7.4)	57.8 (8.1)	63.3 (9.9)	21.15	**<.001**
** Female, *n* (%)**	15 (33)	21 (38)	31 (42)	1.03	.596
** Racial/Ethnic minority, *n* (%)**	13 (28)	16 (29)	11 (15)	4.65	.098
** HF duration, *n* (%)**				4.70	.320
** ≤1 year**	19 (41)	27 (49)	41 (55)		
** 2-4 years**	16 (35)	14 (25)	13 (18)		
** ≥5 years**	11 (24)	14 (25)	20 (27)		
** Prior tracker use (yes), *n* (%)**	11 (24)	15 (27)	40 (54)	14.69	**<.001**
** Intervention group, *n* (%)**	23 (50)	27 (49)	36 (49)	0.02	.990
** Smartphone type, *n* (%)**				2.26	.323
** Android**	30 (65)	32 (58)	38 (51)		
** iOS**	16 (35)	23 (42)	36 (49)		
** Employment status, *n* (%)**				31.62	**<.001**
** Employed**	14 (30)	18 (33)	28 (38)		
** Retired**	6 (13)	11 (20)	33 (45)		
** Disabled**	19 (41)	19 (35)	4 (5)		
** Unemployed**	7 (15)	7 (13)	9 (12)		
**Social determinants of health**					
** Food insecurity (yes), *n* (%)**	17 (37)	17 (31)	0 (0)	31.50	**<.001**
** Housing concern (yes), *n* (%)**	13 (28)	16 (29)	2 (3)	19.83	**<.001**
** Transportation difficulty (yes), *n* (%)**	23 (50)	21 (38)	3 (4)	35.72	**<.001**
** Neighborhood safety concern (yes), *n* (%)**	6 (13)	5 (9)	5 (7)	1.35	.509
**Baseline HF measures**					
** Functional status (KCCQ), *M* (SD)**	50.4 (22.7)	50.6 (23.4)	76.6 (18.2)	48.37	**<.001**
** QoL (KCCQ), *M* (SD)**	35.0 (25.1)	36.2 (22.1)	60.0 (21.3)	42.01	**<.001**
** HF knowledge (AHFKT), *M* (SD)**	79.2 (6.9)	83.5 (8.7)	84.7 (9.9)	15.47	**<.001**
** Self-care (EHFScBS), *M* (SD)**	71.7 (18.9)	73.1 (19.6)	73.2 (18.3)	0.39	.823

Values are *M* (SD) for continuous variables and *n* (%) for categorical variables. *H* from Kruskal-Wallis tests (continuous) or χ^2^ Pearson chi-square tests (categorical). Bold values indicate P < .05.

Abbreviations: AHFKT, Atlanta HF Knowledge Test; EHFScBS, European Heart Failure Self-care Behaviour Scale; HF, heart failure; KCCQ, Kansas City Cardiomyopathy Questionnaire; QoL, quality of life.

A 3-cluster solution was selected, yielding 3 phenotypes that were largely consistent with those identified in our prior work: Engaged Self-Regulators (*n* = 74, 42%), Activated Learners (*n* = 55, 31%), and Challenged Survivors (*n* = 46, 26%).^29^ These labels were established in our prior mixed-methods study, where they reflected overall psychosocial profiles, digital engagement ­behavior, and qualitative insights on engagement quality and ­psychosocial context, rather than longitudinal outcomes. They were retained here because the early derived clusters closely reproduced those previously identified profiles.

The 3-cluster solution was selected based on average silhouette width (0.29), bootstrap stability (mean Jaccard indices: 0.96 across 500 resamples), and interpretability (Figure 1; Figure S1). A 2-cluster solution yielded a marginally higher silhouette width (0.31 vs 0.29), but cross-tabulation showed that it largely combined the Challenged and Activated phenotypes into a single cluster, while preserving the Engaged phenotype. Additionally, the elbow criterion favored 3 clusters, and solutions with *k* ≥ 4 performed worse on all indices. Phenotype assignments were also robust to the early engagement window; clustering on full-period engagement produced nearly identical assignments (96.6% agreement; adjusted Rand index = .90).

**Figure 1:**
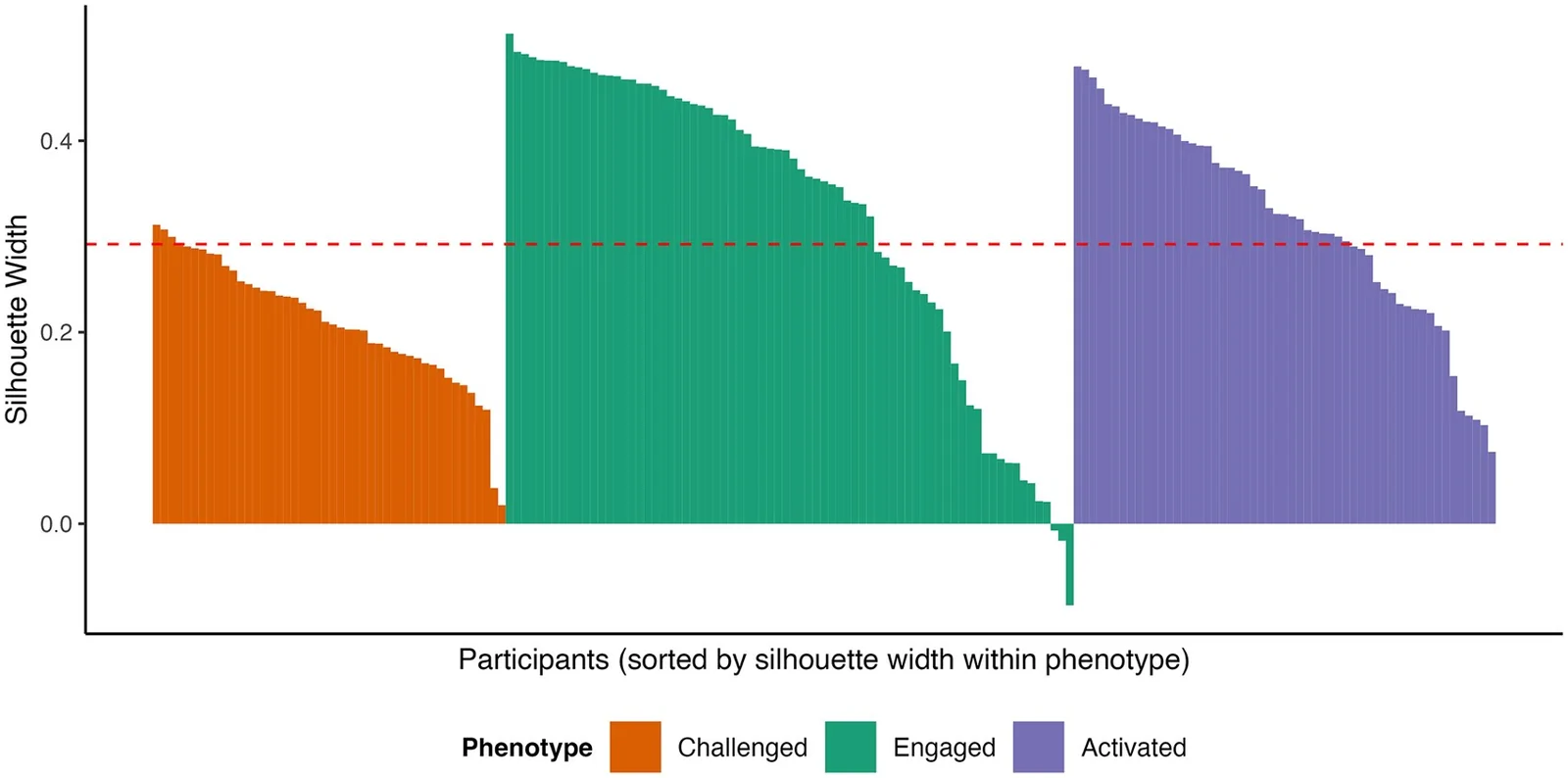
Silhouette plot for the 3-cluster solution.

Phenotypes were characterized by meaningful differences in psychosocial profiles and digital engagement behaviors (Table S4). The Engaged phenotype had the most favorable affective-motivational profile (lowest anxiety and depressed mood, highest motivation), and strongest social resources (92% partnered, 10% financial hardship). The Activated phenotype had the highest anxiety and lowest self-efficacy, but moderate motivation. Notably, despite all having more than a high school education, 95% reported financial hardship. The Challenged phenotype had the lowest motivation and greatest structural disadvantage (76% financial hardship, 0% college educated, 83% single).

### Baseline characteristics by phenotype

There were significant differences between phenotypes in demographics, social determinants, and baseline health status (Table 1). Participants in the Engaged phenotype were older (mean age 63.3), more likely to be retired (45%), and had the highest rate of prior tracker use (54%; all *P* values less than .001). The most striking differences were in social determinants, with food insecurity, housing concerns, and transportation difficulty being reported by 28%-50% of Challenged and Activated participants compared with 0%-4% of Engaged participants (*P* < .001). For baseline health status, the Engaged phenotype had considerably higher functional status and QoL than both other groups (*P* < .001), and HF knowledge was higher among Engaged and Activated participants than Challenged (Table S5). Sex, minority status, HF duration, study group assignment, perceived neighborhood safety, and baseline self-care did not differ across phenotypes.

### Phenotype differences in device engagement

During the initial 4-week period, device engagement was moderate-to-high across all 3 phenotypes (tracker 79%-87%; scale 51%-67% of days). Over the remainder of the study, however, phenotypes differed significantly in device use during weeks 5-24 (tracker: *P* = .03; scale: *P* < .001). The Engaged phenotype sustained the highest use (tracker 77%; scale 59% of days), followed by Activated (tracker 67%; scale 43%) and Challenged (tracker 59%; scale 33%) phenotypes. Tracker use declined approximately 20 percentage points from the initial engagement window in both the Challenged and Activated phenotypes, whereas use among Engaged participants remained relatively stable. In post hoc comparisons, the Engaged phenotype exceeded Challenged on both devices and the Activated phenotype on scale use (all *P* values less than .05), while the Activated and Challenged phenotypes did not differ. Full pairwise comparisons are presented in Table S5.

### EMA affective and motivational states over time

Phenotypes differed significantly across all EMA affective-motivational measures (all *P* values less than .001; Table 2), with no significant EMA wave or interaction effects, indicating that between-phenotype differences in affective-motivational states persisted over time. Across all timepoints, Engaged participants consistently reported lower anxiety, lower depressed mood, and higher motivation than both other groups (*P* < .05; Figure 2). The Challenged and Activated phenotypes did not significantly differ from each other on any affective-motivational measure. Although differences were not statistically significant, Activated participants reported descriptively higher motivation than Challenged participants across time. Descriptives and complete pairwise contrasts are reported in Tables S6 and S7. Complete-case sensitivity analyses (*n* = 121) showed the same pattern, with phenotype effects remaining significant for all 3 measures (all *P* values less than .005; Table S8).

**Figure 2:**
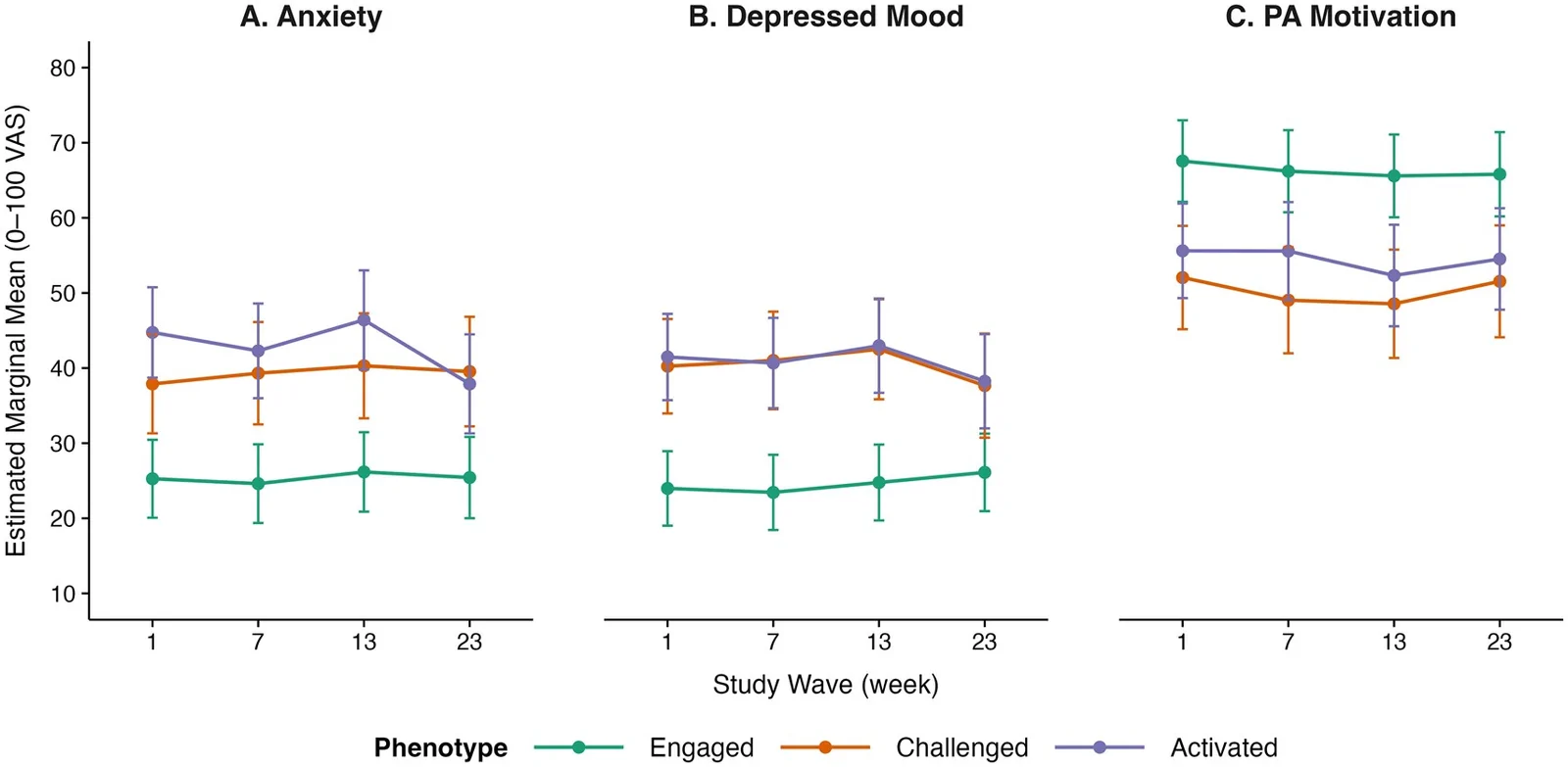
Estimated marginal means of momentary affective-motivational measures by phenotype across study waves. Error bars represent 95% confidence intervals from random-intercept linear mixed-effects models.

**Table 2:** Linear mixed-effects model results for momentary psychological states by phenotype.

	Anxiety	Depressed mood	PA motivation
**Type III fixed effects**			
** Phenotype, *F* (*P*)**	13.57 **(<.001)**	14.58 **(<.001)**	8.90 **(<.001)**
** Phenotype, partial *η* ^2^ (95% CI)**	.13 (.05-.23)	.14 (.06-.24)	.09 (.02-.18)
** Study wave, *F* (*P*)**	1.48 (.22)	1.14 (.33)	1.55 (.20)
** Phenotype × wave, *F* (*P*)**	1.17 (.32)	0.96 (.45)	0.31 (.93)
**Pairwise contrasts: week 1, *b* (*P*)**			
** Engaged-Challenged**	−12.63 **(.009)**	−16.28 **(<.001)**	15.51 **(.002)**
** Engaged-Activated**	−19.48 **(<.001)**	−17.50 **(<.001)**	11.95 **(.014)**
** Challenged-Activated**	−6.86 (.286)	−1.23 (.957)	−3.56 (.733)
**Pairwise contrasts: week 23, *b* (*P*)**			
** Engaged-Challenged**	−14.11 **(.007)**	−11.56 **(.024)**	14.26 **(.008)**
** Engaged-Activated**	−12.47 **(.012)**	−12.13 **(.010)**	11.28 **(.032)**
** Challenged-Activated**	1.64 (.942)	−0.58 (.992)	−2.98 (.829)

Bold values indicate P < .05.

### Phenotype membership and patient-reported HF outcomes

Fixed effects are presented in Table 3, estimated marginal means in Table 4, and phenotype trajectories in Figure 3. For functional status and QoL, all 3 phenotypes improved significantly over time (*b* = 0.14 and 0.36 points/week, respectively; both *P* ≤ .002), corresponding to approximate 24-week increases of 3.3 and 8.7 points. The Engaged phenotype had functional status and QoL scores 20-23 points higher than both other phenotypes across timepoints (*P* < .001), with no significant phenotype × time interactions. The Challenged and Activated groups did not differ from each other (*P* > .05). Among covariates, female sex was associated with lower functional status (*P* = .037), and intervention group was associated with lower QoL (*P* = .049).

**Figure 3:**
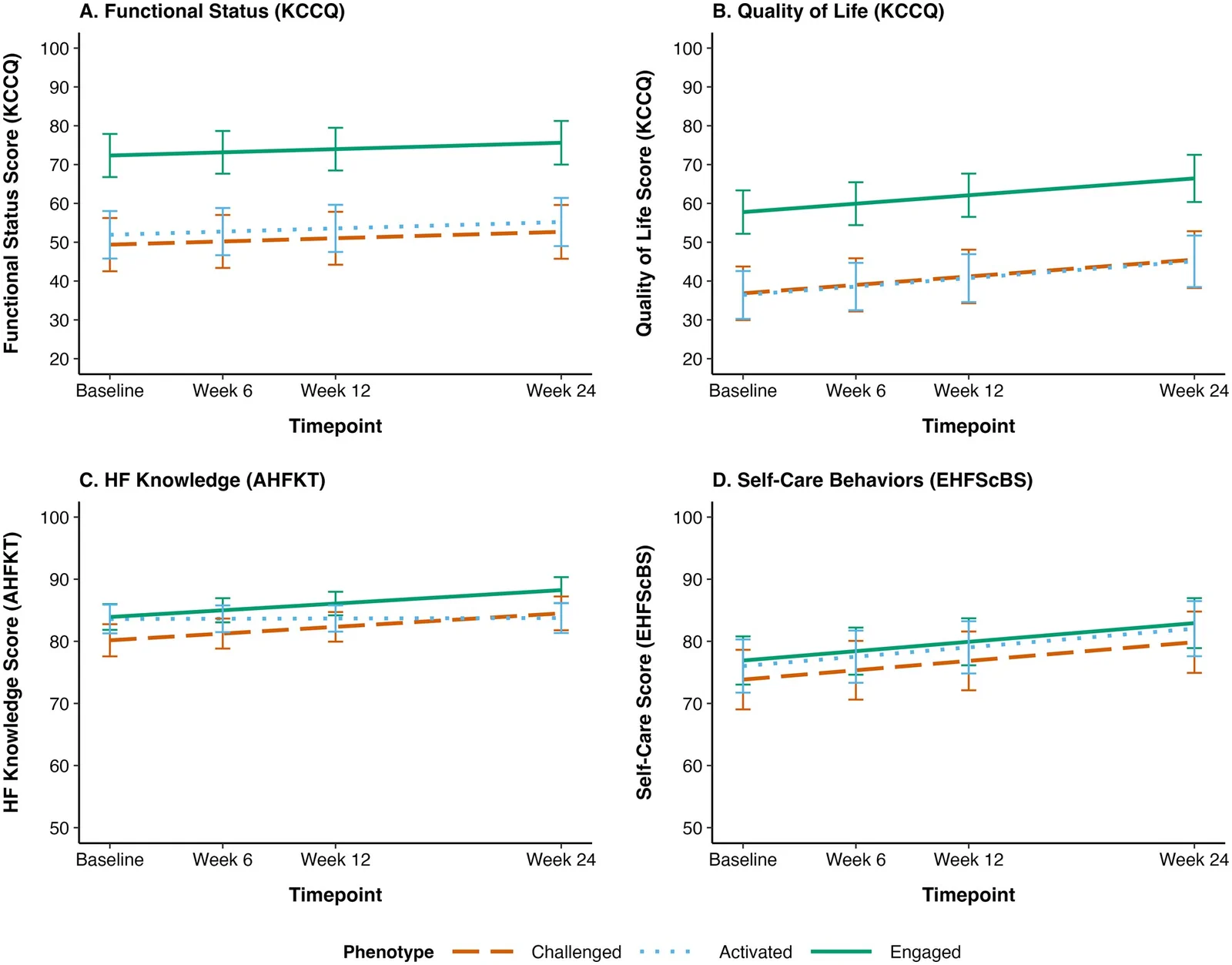
Estimated marginal means of HF health outcomes by phenotype across study timepoints. Error bars represent 95% confidence intervals from covariate-adjusted linear mixed-effects models. Covariates include age (centered), sex, minority status, study group assignment, and prior tracker ownership. For Functional Status (A), QoL (B), and Self-Care Behaviors (D), estimates are from main-effects models; for HF Knowledge (C), estimates are from the phenotype × time interaction model. Abbreviations: HF, heart failure; QoL, quality of life.

**Table 3:** Linear mixed-effects model results for HF health outcomes by phenotype (*N *= 175).

Predictor	Functional status		QoL		HF knowledge		Self-care	
	*b* (95% CI)	*P*	*b* (95% CI)	*P*	*b* (95% CI)	*P*	*b* (95% CI)	*P*
**Type III omnibus tests**								
** Phenotype, *F* (*P*)**	18.94 (<.001)		18.01 (<.001)		3.15 (.045)		0.57 (.566)	
** Phenotype, partial *η* ^^2^^ (95% CI)**	.19 (.09 to .28)		.18 (.08 to .27)		.04 (.00 to .10)		.01 (.00 to .04)	
** Phenotype × Time, *F (*P*)***	–		–		5.23 (.006)		–	
** Phenotype × Time, partial *η* ^^2^^ (95% CI)**	–		–		.06 (.01 to .14)		–	
**Fixed effects**								
** Intercept**	75.79 (68.39 to 83.19)	<.001	63.85 (56.41 to 71.29)	<.001	83.38 (80.70 to 86.06)	<.001	72.95 (67.81 to 78.09)	<.001
** Time (weeks)**	0.14 (0.05 to 0.22)	.002	0.36 (0.22 to 0.50)	<.001	0.18 (0.11 to 0.25)	<.001	0.25 (0.15 to 0.35)	<.001
** Challenged (vs engaged)**	−22.96 (−31.28 to −14.63)	<.001	−20.92 (−29.28 to −12.55)	<.001	−3.75 (−6.93 to −0.57)	.022	−3.07 (−8.80 to 2.66)	.295
** Activated (vs Engaged)**	−20.42 (−28.14 to −12.70)	<.001	−21.35 (−29.10 to −13.59)	<.001	−0.33 (−3.28 to 2.63)	.829	−0.89 (−6.21 to 4.42)	.742
** Age (centered)**	−0.11 (−0.47 to 0.25)	.556	0.09 (−0.28 to 0.46)	.631	−0.01 (−0.14 to 0.11)	.862	0.01 (−0.24 to 0.26)	.943
** Female (vs male)**	−6.87 (−13.28 to −0.46)	.037	−3.92 (−10.36 to 2.53)	.235	1.41 (−0.81 to 3.64)	.215	−3.87 (−8.28 to 0.55)	.088
** Minority (vs NH White)**	−6.52 (−14.07 to 1.02)	.092	−5.86 (−13.44 to 1.72)	.132	−4.05 (−6.67 to −1.42)	.003	3.16 (−2.06 to 8.38)	.237
** Intervention (vs control)**	−3.46 (−9.62 to 2.71)	.273	−6.26 (−12.45 to −0.07)	.049	1.15 (−0.98 to 3.29)	.292	2.45 (−1.80 to 6.70)	.260
** Prior tracker use (vs no)**	10.04 (3.34 to 16.75)	.004	3.77 (−2.96 to 10.50)	.274	2.56 (0.25 to 4.88)	.032	6.17 (1.57 to 10.77)	.009
** Time × Challenged**	–	–	–	–	0.00 (−0.12 to 0.12)	.995	–	–
** Time × Activated**	–	–	–	–	−0.17 (−0.29 to −0.06)	.003	–	–
**Random effects**								
** Structure**	RI only		RI + RS		RI + RS		RI only	
** ICC**	.865		.789		.716		.606	
** σ^2^ intercept (SD)**	403.87 (20.10)		380.17 (19.50)		51.59 (7.18)		169.80 (13.03)	
** σ^2^ slope (SD)**	–		0.36 (0.60)		0.023 (0.15)		–	
** Intercept-slope *r***	–		−0.05		−0.39		–	
** σ^2^ residual (SD)**	87.68 (9.36)		138.65 (11.78)		20.57 (4.54)		111.30 (10.55)	
**Model information**								
** Participants**	175		175		175		175	
** Observations**	637		637		626		623	
** Mean obs/participant**	3.64		3.64		3.58		3.56	

Omnibus *P*-values are unadjusted; Benjamini-Hochberg–adjusted *P*-values are reported in text.

Abbreviations: HF, heart failure; ICC, intraclass correlation coefficient from unconditional models; Intercept-slope *r*, correlation between random intercept and random slope; RI, random intercept; RS, random slope of time; σ^2^, variance estimate with standard deviation in parentheses.

**Table 4:** Estimated marginal means and pairwise comparisons for HF health outcomes by phenotype (*N* = 175).

	Functional status	QoL	HF knowledge				Self-care
			Baseline	Week 6	Week 12	Week 24	
**EMM (95% CI)**							
** Challenged**	50.7 (43.9 to 57.5)	40.4 (33.5 to 47.3)	80.2 (77.6 to 82.8)	81.2 (78.8 to 83.7)	82.3 (79.9 to 84.7)	84.5 (81.8 to 87.2)	76.3 (71.6 to 81.0)
** Activated**	53.3 (47.2 to 59.3)	40.0 (33.8 to 46.1)	83.6 (81.3 to 85.9)	83.6 (81.5 to 85.8)	83.7 (81.6 to 85.8)	83.8 (81.3 to 86.2)	78.5 (74.3 to 82.7)
** Engaged**	73.7 (68.2 to 79.2)	61.3 (55.8 to 66.9)	83.9 (81.8 to 86.0)	85.0 (83.1 to 86.9)	86.1 (84.2 to 88.0)	88.2 (86.1 to 90.3)	79.4 (75.6 to 83.1)
**Pairwise comparisons, *P***							
** Engaged vs Challenged**	**<.001**	**<.001**	.057	.037	.032	.069	.546
** Engaged vs Activated**	**<.001**	**<.001**	.975	.592	.187	**.013**	.942
** Challenged vs Activated**	.816	.994	.090	.247	.629	.902	.732

Abbreviations: HF, heart failure; QoL, quality of life. Bold values indicate P < .05.

Heart failure knowledge showed diverging phenotype trajectories (phenotype × time, adjusted *P* = .008). Although unadjusted baseline comparisons differed across phenotypes (Table 1), adjusted pairwise differences in HF knowledge at baseline did not remain significant after correcting for multiple comparisons (*P* = .057). However, while the Engaged and Challenged phenotypes improved at similar rates over time (0.18 points/week each, approximately 4 points over 24 weeks), the Activated participants showed no meaningful improvement in knowledge scores. Both slopes were significantly steeper than Activated (*P* = .009 and .03, respectively). By the final week of the study, Engaged participants had significantly higher knowledge scores than Activated (*P* = .013); no other differences at week 24 reached significance. Racial and ethnic minority status was associated with lower HF knowledge across timepoints (*P* = .003). Self-reported self-care improved over time across all groups (*b* = 0.25 points/week, *P* < .001) but did not differ by phenotype (*P* > .05). In 3 of the 4 outcome models, prior tracker use was associated with better outcomes, including higher functional status (*P* = .004), HF knowledge (*P* = .032), and self-care (*P* = .009).

Pattern-mixture models and tipping point analyses supported these findings under potential departures from missing at random (MAR; Tables S10 and S11). No significant dropout effects emerged for any outcome, and a 3-way model confirmed that the Activated phenotype’s flat knowledge trajectory was not an artifact of differential attrition. Phenotype differences in functional status and QoL remained robust under increasingly pessimistic dropout assumptions. The most sensitive finding was the time effect for functional status, which tipped at δ = −8.5 (1.7 times the MCID), indicating that dropouts would need to score 8.5 points below MAR-predicted values for the observed improvement over time to no longer reach statistical significance. Full sensitivity procedures and results are included in Supplementary Material S1.

## Discussion

### Principal findings

Using baseline psychosocial characteristics and the first 4 weeks of device engagement data from a 6-month digital intervention for HF, we identified 3 psychosocial-behavioral phenotypes that were significantly associated with subsequent device engagement and longitudinal patient-reported HF outcomes. The early derived phenotypes closely matched those previously identified using full-period engagement in our prior work, suggesting that these subgroups are identifiable within the first month of a digital intervention. Importantly, these phenotypes captured multidimensional constellations of affective-motivational profiles, social and structural context, and early digital engagement behavior.

This study extends our prior phenotyping work in several ways. While our prior phenotypes incorporated device engagement across the full intervention period and therefore could only be identified retrospectively, the present analysis reproduced the 3 phenotypes using early engagement data and demonstrated associations with subsequent digital engagement during weeks 5-24. Between-phenotype differences in affective-motivational states also persisted across EMA waves over the 6-month intervention, and phenotype membership was associated with persistent differences in functional status and QoL, and divergent HF knowledge trajectories, but not self-care behaviors. This work aligns with emerging precision health approaches that call for the incorporation of behavioral, social, and environmental determinants alongside biological factors in chronic disease management.^43–45^ Together, these findings advance understanding of heterogeneity in DHI engagement and outcomes, and provide a foundation for prospectively testing phenotype-informed tailoring of DHIs for adults with HF.

### Phenotype differences and structural disadvantage

The phenotypes were characterized by marked differences in social and structural context. Although Challenged and Activated phenotypes reported similarly elevated psychological distress and financial hardship, the Challenged group experienced the greatest burden of unmet basic needs, lowest educational attainment and social connectedness, and the lowest subsequent device use. These findings align with broader evidence demonstrating that adverse social and structural determinants constrain digital adoption and sustained engagement.^46^^,^^47^ Lower engagement in the Challenged phenotype may therefore reflect competing demands associated with economic and social hardship, rather than disengagement as an individual choice. For individuals in this phenotype, digital tools alone may be insufficient to overcome barriers such as food insecurity, housing instability, and transportation needs, suggesting that future DHIs could test integration of social service referrals and human support.

Prior tracker ownership was also more common among Engaged participants and was associated with better outcomes in several models, suggesting that prior digital experience may be an important marker of readiness for DHI engagement. Rather than assuming uniform technology readiness and literacy, early assessment of digital experience may help identify individuals who may benefit from additional onboarding or digital literacy support.^48^

### Temporal stability of affective and motivational states

Between-phenotype differences in anxiety, depressed mood, and motivation persisted across 4 EMA waves over 6 months, suggesting the phenotypes captured relatively enduring affective-motivational profiles. This aligns with prior research showing that, while EMAs can capture substantial within-person variability,^49^ daily affect (eg, self-reported moods) is generally stable when measured over weeks or months.^50^^,^^51^ Further, the AIM-ACT framework posits that affective and motivational responses shape how individuals engage with digital interventions,^24^ which is concretely exemplified by these findings—for example, the Engaged phenotype’s lower distress and higher motivation may reflect a more favorable starting point for digital engagement, whereas persistently elevated distress among Challenged and Activated participants may represent a potential barrier. These findings suggest that early EMA measures may help identify affective-motivational profiles relevant to engagement and warrant evaluation as potential tailoring variables for adaptive and just-in-time digital interventions.^52^ Prospective studies are needed to determine whether these measures can effectively guide intervention adaptation and improve subsequent DHI engagement and outcomes.

### HF patient-reported outcomes across phenotypes

All 3 phenotypes improved significantly in functional status, QoL, and self-care over 6 months, with similar rates of change despite considerable differences in psychosocial characteristics and device engagement. However, the Engaged phenotype began with substantially higher functional status and QoL scores at baseline and maintained differences of 20-23 points throughout follow-up, a difference of approximately 4-5 times the minimal clinically important difference for the KCCQ.^53^^,^^54^ Since KCCQ domains are independently associated with subsequent mortality and hospitalization, these persistent between-phenotype differences may be clinically meaningful.^55^ Self-care was the only outcome that did not differ by phenotype, consistent with the baseline pattern (Table 1).

The similar trajectories in improvement for these outcomes despite different levels of device engagement do not support a simple linear dose-response relationship between engagement and outcomes. Although the Challenged phenotype had the lowest device engagement, they still used the activity tracker on 62% and scale on 36% of days across the full study period, suggesting that outcomes can improve even in the context of low-moderate digital engagement. The phenotypes may also partly reflect underlying health status, as functional status and QoL were already substantially higher in Engaged phenotype at baseline. This aligns with the healthy adherer effect, in which individuals in better health may be more likely to engage with health interventions.^56^ The persistence of large between-phenotype differences suggests that digital interventions alone may be insufficient to reduce preexisting disparities when psychosocial and structural barriers remain unaddressed.^48^

### Divergent HF knowledge trajectories

Heart failure knowledge trajectories presented a more nuanced pattern. While the Engaged and Challenged phenotypes improved modestly, the Activated phenotype showed no meaningful change. This finding is notable given that Activated participants had high educational attainment and moderate digital engagement. One potential explanation is that elevated anxiety and lower self-efficacy may interfere with cognitive integration of health information, limiting translation of information into retained knowledge.^24^^,^^25^ Qualitative findings from our prior mixed-methods study offer an alternative interpretation. Activated Learners described using digital tools to develop practical understanding of symptom patterns and relationships between their symptom experiences and device feedback.^29^ Although these accounts cannot be generalized to the full phenotype, they raise the possibility that experiential learning through digital tools may not be fully captured by standardized HF knowledge measures.

Racial and ethnic minority status was independently associated with lower HF knowledge across time, with minority participants scoring approximately 4 points lower than non-Hispanic Whites. This finding is consistent with documented disparities in HF knowledge and health literacy and highlights the importance of ensuring that educational components of digital interventions are accessible and culturally responsive.^57^^,^^58^

### Implications for precision digital health interventions

Our findings advance precision digital health by demonstrating that multidimensional psychosocial-behavioral phenotypes can be identified early in digital intervention delivery and are meaningfully associated with subsequent engagement and longitudinal outcomes in adults with HF. These phenotypes provide testable hypotheses for future studies evaluating whether phenotype-informed adaptation can improve DHI engagement and effectiveness. One potential pathway for operationalizing this approach is a phenotype-informed multimodal adaptive intervention (MADI) framework, which integrates human-delivered and digital components across slow and fast tailoring timescales.^59^ At the slow timescale (weeks to months), early phenotype identification could guide initial intervention intensity, modality, and level of human support. At the fast timescale (hours to days), ongoing data on intervention engagement and dynamic affective-motivational states could trigger dynamic, just-in-time nudges. In this way, relatively stable psychosocial-behavioral characteristics could inform the intervention’s overall structure, while more proximal behavior and contextual signals guide moment-to-moment support.

Applied to our sample, this framework suggests different starting configurations for each phenotype. Future phenotype-informed interventions could test whether the Challenged phenotype would benefit from higher-touch human support and assistance addressing social and digital barriers; Activated from coaching and support for addressing social and affective barriers such as anxiety; and whether the Engaged phenotype can be supported with more autonomous, self-directed digital tools. This stratified approach aligns with the precision health paradigm and the AIM-ACT framework’s emphasis on matching intervention components to psychosocial and behavioral context.^24^^,^^59^ Whether phenotype-informed MADI approaches improve sustained engagement, outcomes, or disparities in cardiovascular digital health remains to be established and warrants prospective evaluation. Future studies should test whether early phenotype-informed adaptation improves engagement and outcomes compared with nontailored digital interventions.

### Limitations

This study has several limitations. First, the sample was majority non-Hispanic White, English-speaking older adults, so findings may not be representative of the broader HF population. Second, psychosocial characteristics and HF outcomes were self-reported; future studies incorporating objective clinical endpoints may provide additional insight into associations between phenotypes and health outcomes. Third, outcome missingness differed by phenotype and was greatest among Challenged participants. Although pattern-mixture models and tipping point analyses supported robustness of findings to differential attrition, the MAR assumption cannot be fully verified. Fourth, phenotypes were derived from baseline psychosocial characteristics and the first 4 weeks of device engagement. Although these inputs preceded subsequent engagement and outcome assessments, the analysis was observational and cannot be interpreted as causal. Fifth, although the three-cluster solution demonstrated high bootstrap stability and reproduced the prior phenotype structure, the modest average silhouette width (0.29) indicates limited separation between clusters. The phenotypes should therefore be interpreted as data-driven groupings rather than discrete clinical categories. Lastly, we used device syncing as a proxy for use of the digital tool, which does not capture the quality or subjective experience of engagement.

## Conclusion

Early psychosocial-behavioral phenotyping identified subgroups characterized by meaningful differences in psychosocial characteristics, social and structural context, subsequent digital engagement, and longitudinal HF outcomes. Phenotypes were associated with persistent disparities in HF-related functional status and QoL and marked differences in social determinants. These findings demonstrate the potential value of psychosocial-behavioral phenotyping to understand heterogeneity in digital interventions and provide a foundation for prospectively testing whether phenotype-informed adaptation can improve engagement and outcomes.

## Supplementary Material

kaag066_Supplementary_Data

## Open science transparency statements

### Study registration

This study was not formally registered. The parent randomized controlled trial from which this sec­ondary analysis draws data is registered at ClinicalTrials.gov (NCT05056129).

### Analytic plan preregistration

The analysis plan was not for­mally preregistered.

### Data Availability

Deidentified data from this study are not available in a public archive. Deidentified data from this study will be made available (as allowable according to institutional IRB standards) by emailing the corresponding author.

### Analytic code availability

Analytic code used to conduct the analyses presented in this study is not available in a public archive. Code may be made available by emailing the corre­sponding author.

### Materials availability

Materials used to conduct the parent trial, including the trial protocol, intervention components, and survey instruments, are described in the published trial protocol.^30^

## References

[1] Fonarow G , Ahmad F, Ahmad T, et al HF STATS 2025: heart failure epidemiology and outcomes statistics: an updated 2025 report from the Heart Failure Society of America. J Card Fail. 2026;32:439-498. 10.1016/j.cardfail.2025.07.007 40987671

[2] Salah HM , Minhas AMK, Khan MS, et al Causes of hospitalization in the USA between 2005 and 2018. Eur Heart J Open. 2021;1:oeab001. 10.1093/ehjopen/oeab001 PMC9242058 35919090

[3] Johansson I , Joseph P, Balasubramanian K, et al; G-CHF Investigators. Health-related quality of life and mortality in heart failure: the global congestive heart failure study of 23 000 patients from 40 countries. Circulation. 2021;143:2129-2142. 10.1161/CIRCULATIONAHA.120.050850 33906372

[4] Riegel B , Moser DK, Buck HG, et al; on behalf of the American Heart Association Council on Cardiovascular and Stroke Nursing; Council on Peripheral Vascular Disease; and Council on Quality of Care and Outcomes Research. Self-care for the prevention and management of cardiovascular disease and stroke. J Am Heart Assoc. 2017;6:e006997. 10.1161/JAHA.117.006997

[5] Jaarsma T , Hill L, Bayes-Genis A, et al Self-care of heart failure patients: practical management recommendations from the Heart Failure Association of the European Society of Cardiology. Eur J Heart Fail. 2021;23:157-174. 10.1002/ejhf.2008 PMC8048442 32945600

[6] Riegel B , Jaarsma T, Strömberg A. A middle-range theory of self-care of chronic illness. ANS Adv Nurs Sci. 2012;35:194-204. 10.1097/ANS.0b013e318261b1ba 22739426

[7] Seid SS , Amendoeira J, Ferreira MR. Self-care and quality of life among adult patients with heart failure: scoping review. SAGE Open Nurs. 2023;9:23779608231193719. 10.1177/23779608231193719 PMC10413905 37576941

[8] Lee CS , Bidwell JT, Paturzo M, et al Patterns of self-care and clinical events in a cohort of adults with heart failure: 1 year follow-up. Heart Lung. 2018;47:40-46. 10.1016/j.hrtlng.2017.09.004 PMC6497061 29054487

[9] Marti CN , Georgiopoulou VV, Giamouzis G, et al Patient-reported selective adherence to heart failure self-care recommendations: a prospective cohort study: the Atlanta Cardiomyopathy Consortium. Congest Heart Fail. 2013;19:16-24. 10.1111/j.1751-7133.2012.00308.x PMC3519965 22958604

[10] Madujibeya I , Lennie T, Aroh A, Chung ML, Moser D. Measures of engagement with mHealth interventions in patients with heart failure: scoping review. JMIR Mhealth Uhealth. 2022;10:e35657. 10.2196/35657 PMC9446141 35994345

[11] Azizi Z , Broadwin C, Islam S, et al Digital health interventions for heart failure management in underserved rural areas of the United States: a systematic review of randomized trials. J Am Heart Assoc. 2024;13:e030956. 10.1161/JAHA.123.030956 PMC10926837 38226517

[12] Kitsiou S , Vatani H, Paré G, et al Effectiveness of mobile health technology interventions for patients with heart failure: systematic review and meta-analysis. Can J Cardiol. 2021;37:1248-1259. 10.1016/j.cjca.2021.02.015 33667616

[13] Liu S , Li J, Wan DY, et al Effectiveness of eHealth self-management interventions in patients with heart failure: systematic review and meta-analysis. J Med Internet Res. 2022;24:e38697. 10.2196/38697 PMC9555330 36155484

[14] Cole-Lewis H , Ezeanochie N, Turgiss J. Understanding health behavior technology engagement: pathway to measuring digital behavior change interventions. JMIR Form Res. 2019;3:e14052. 10.2196/14052 PMC6813486 31603427

[15] Perski O , Blandford A, West R, Michie S. Conceptualising engagement with digital behaviour change interventions: a systematic review using principles from critical interpretive synthesis. Transl Behav Med. 2017;7:254-267. 10.1007/s13142-016-0453-1 PMC5526809 27966189

[16] Eysenbach G. The law of attrition. J Med Internet Res. 2005;7:e11. 10.2196/jmir.7.1.e11 PMC1550631 15829473

[17] Meyerowitz-Katz G , Ravi S, Arnolda L, Feng X, Maberly G, Astell-Burt T. Rates of attrition and dropout in app-based interventions for chronic disease: systematic review and meta-analysis. J Med Internet Res. 2020;22:e20283. 10.2196/20283 PMC7556375 32990635

[18] Gingele AJ , Steiner B, Zippel-Schultz B, Brunner-La Rocca HP. Adherence to eHealth interventions among patients with heart failure: scoping review. JMIR Mhealth Uhealth. 2025;13:e63409. 10.2196/63409 PMC12227154 40577640

[19] Pratap A , Neto EC, Snyder P, et al Indicators of retention in remote digital health studies: a cross-study evaluation of 100,000 participants. NPJ Digit Med. 2020;3:21. 10.1038/s41746-020-0224-8 PMC7026051 32128451

[20] Deshpande N , Arora VM, Vollbrecht H, Meltzer DO, Press V. eHealth literacy and patient portal use and attitudes: cross-sectional observational study. JMIR Hum Factors. 2023;10:e40105. 10.2196/40105 PMC9919456 36705947

[21] Chisolm RB , Tomori C, Spaulding EM. The impact of digital health literacy on patient adherence, self-management, and follow-up care: an integrative review. Telemed J E Health. 2026;32:256-268. 10.1177/15305627251401713 41355792

[22] Veinot TC , Mitchell H, Ancker JS. Good intentions are not enough: how informatics interventions can worsen inequality. J Am Med Inform Assoc. 2018;25:1080-1088. 10.1093/jamia/ocy052 PMC7646885 29788380

[23] Veinot TC , Clarke PJ, Romero DM, et al Equitable research PRAXIS: a framework for health informatics methods. Yearb Med Inform. 2022;31:307-316. 10.1055/s-0042-1742542 PMC9719773 36463889

[24] Nahum-Shani I , Shaw SD, Carpenter SM, Murphy SA, Yoon C. Engagement in digital interventions. Am Psychol. 2022;77:836-852. 10.1037/amp0000983 PMC9481750 35298199

[25] Nahum-Shani I , Yoon C. Towards the science of engagement with digital interventions. Curr Dir Psychol Sci. 2024;33:239-246. 10.1177/09637214241254328 PMC11567151 39552747

[26] Kim MT , Radhakrishnan K, Heitkemper EM, Choi E, Burgermaster M. Psychosocial phenotyping as a personalization strategy for chronic disease self-management interventions. Am J Transl Res. 2021;13:1617-1635. PMC8014371 33841684

[27] Burgermaster M , Rodriguez VA. Psychosocial-behavioral phenotyping: a novel precision health approach to modeling behavioral, psychological, and social determinants of health using machine learning. Ann Behav Med. 2022;56:1258-1271. 10.1093/abm/kaac012 PMC9672350 35445699

[28] Yardley L , Spring BJ, Riper H, et al Understanding and promoting effective engagement with digital behavior change interventions. Am J Prev Med. 2016;51:833-842. 10.1016/j.amepre.2016.06.015 27745683

[29] Tunis R , Clifford N, West E, Heitkemper E, Burgermaster M, Radhakrishnan K. Developing psychosocial phenotypes to understand engagement with digital health technologies for heart failure. NPJ Digit Med. 2026;9:420. 10.1038/s41746-026-02571-z PMC13230796 41935162

[30] Radhakrishnan K , Julien C, O’Hair M, et al Sensor-controlled digital game for heart failure self-management: protocol for a randomized controlled trial. JMIR Res Protoc. 2023;12:e45801. 10.2196/45801 PMC10209796 37163342

[31] Radhakrishnan K , DeMain AS, Julien C, et al A sensor-controlled digital game for heart failure self-care based on behavioral change frameworks. In: *2022* IEEE *10th International Conference on Serious Games and Applications for Health (SeGAH)*. IEEE; 2022. 10.1109/segah54908.2022.9978594 PMC10564567 37817860

[32] von Elm E , Altman DG, Egger M, Pocock SJ, Gøtzsche PC, Vandenbroucke JP; STROBE Initiative. Strengthening the reporting of observational studies in epidemiology (STROBE) statement: guidelines for reporting observational studies. BMJ. 2007;335:806-808. 10.1136/bmj.39335.541782.AD PMC2034723 17947786

[33] Riegel B , Lee CS, Dickson VV, Carlson B. An update on the self-care of heart failure index. J Cardiovasc Nurs. 2009;24:485-497. 10.1097/JCN.0b013e3181b4baa0 PMC2877913 19786884

[34] Goevaerts WF , Tenbült-Van Limpt NCCW, Goossen SA, et al Long-term lifestyle monitoring adherence in patients after cardiac intervention: a prospective observational trial. Eur Heart J Digit Health. 2026;7:ztag016. 10.1093/ehjdh/ztag016 PMC12930194 41743190

[35] Green CP , Porter CB, Bresnahan DR, Spertus JA. Development and evaluation of the Kansas city cardiomyopathy questionnaire: a new health status measure for heart failure. J Am Coll Cardiol. 2000;35:1245-1255. 10.1016/S0735-1097(00)00531-3 10758967

[36] Jaarsma T , Arestedt KF, Mårtensson J, Dracup K, Strömberg A. The European Heart Failure Self-care Behaviour scale revised into a nine-item scale (EHFScB-9): a reliable and valid international instrument. Eur J Heart Fail. 2009;11:99-105. 10.1093/eurjhf/hfn007 19147463

[37] Butts B , Higgins M, Dunbar S, Reilly C. The third time’s a charm: psychometric testing and update of the Atlanta Heart Failure Knowledge Test. J Cardiovasc Nurs. 2018;33:13-21. 10.1097/JCN.0000000000000413 PMC5671919 28481824

[38] Kaufman L , Rousseeuw P. Finding Groups in Data: An Introduction to Cluster Analysis. John Wiley & Sons; 1990.

[39] Rousseeuw PJ. Silhouettes: a graphical aid to the interpretation and validation of cluster analysis. J Comput Appl Math. 1987;20:53-65. 10.1016/0377-0427(87)90125-7

[40] Hennig C. Cluster-wise assessment of cluster stability. Comput Stat Data Anal. 2007;52:258-271. 10.1016/j.csda.2006.11.025

[41] Benjamini Y , Hochberg Y. Controlling the false discovery rate: a practical and powerful approach to multiple testing. J R Stat Soc Series B. 1995;57:289-300. 10.1111/j.2517-6161.1995.tb02031.x

[42] R Core Team. R: A Language and Environment for Statistical Computing. R Foundation for Statistical Computing; 2023.

[43] Hekler E , Tiro JA, Hunter CM, Nebeker C. Precision health: the role of the social and behavioral sciences in advancing the vision. Ann Behav Med. 2020;54:805-826. 10.1093/abm/kaaa018 PMC7646154 32338719

[44] Gambhir SS , Ge TJ, Vermesh O, Spitler R. Toward achieving precision health. Sci Transl Med. 2018;10:eaao3612. 10.1126/scitranslmed.aao3612 PMC5985668 29491186

[45] Viana JN , Edney S, Gondalia S, et al Trends and gaps in precision health research: a scoping review. BMJ Open. 2021;11:e056938. 10.1136/bmjopen-2021-056938 PMC8547511 34697128

[46] Kim DS , Eltahir AA, Ngo S, Rodriguez F. Bridging the gap: how accounting for social determinants of health can improve digital health equity in cardiovascular medicine. Curr Atheroscler Rep. 2024;27:9. 10.1007/s11883-024-01249-9 PMC12499349 39576395

[47] Richardson S , Lawrence K, Schoenthaler AM, Mann D. A framework for digital health equity. NPJ Digit Med. 2022;5:119. 10.1038/s41746-022-00663-0 PMC9387425 35982146

[48] Western MJ , Smit ES, Gültzow T, et al Bridging the digital health divide: a narrative review of the causes, implications, and solutions for digital health inequalities. Health Psychol Behav Med. 2025;13:2493139. 10.1080/21642850.2025.2493139 PMC12020140 40276490

[49] Shiffman S , Stone AA, Hufford MR. Ecological momentary assessment. Annu Rev Clin Psychol. 2008;4:1-32. 10.1146/annurev.clinpsy.3.022806.091415 18509902

[50] Hudson NW , Lucas RE, Donnellan MB. Day-to-day affect is surprisingly stable: a two-year longitudinal study of well-being. Soc Psychol Personal Sci. 2017;8:45-54. 10.1177/1948550616662129 PMC5726280 29238453

[51] Cranford JA , Shrout PE, Iida M, Rafaeli E, Yip T, Bolger N. A procedure for evaluating sensitivity to within-person change: can mood measures in diary studies detect change reliably? Pers Soc Psychol Bull. 2006;32:917-929. 10.1177/0146167206287721 PMC2414486 16738025

[52] Nahum-Shani I , Hekler EB, Spruijt-Metz D. Building health behavior models to guide the development of just-in-time adaptive interventions: a pragmatic framework. Health Psychol. 2015;34S:1209-1219. 10.1037/hea0000306 PMC4732268 26651462

[53] Spertus JA , Jones PG, Butler J, Arnold SV. Minimally important Kansas city cardiomyopathy questionnaire changes across the spectrum of heart failure severity. JACC Heart Fail. 2025;13:102587. 10.1016/j.jchf.2025.102587 40908080

[54] Butler J , Khan MS, Mori C, et al Minimal clinically important difference in quality of life scores for patients with heart failure and reduced ejection fraction. Eur J Heart Fail. 2020;22:999-1005. 10.1002/ejhf.1810 32239794

[55] Pokharel Y , Khariton Y, Tang Y, et al Association of serial Kansas city cardiomyopathy questionnaire assessments with death and hospitalization in patients with heart failure with preserved and reduced ejection fraction: a secondary analysis of 2 randomized clinical trials. JAMA Cardiol. 2017;2:1315-1321. 10.1001/jamacardio.2017.3983 PMC5814994 29094152

[56] Haynes SC , Tancredi DJ, Tong K, et al; Better Effectiveness After Transition-Heart Failure (BEAT-HF) Research Group. Association of adherence to weight telemonitoring with health care use and death: a secondary analysis of a randomized clinical trial. JAMA Netw Open. 2020;3:e2010174. 10.1001/jamanetworkopen.2020.10174 PMC7352152 32648924

[57] Chaudhry SI , Herrin J, Phillips C, et al Racial disparities in health literacy and access to care among patients with heart failure. J Card Fail. 2011;17:122-127. 10.1016/j.cardfail.2010.09.016 PMC3053061 21300301

[58] Cajita MI , Cajita TR, Han HR. Health literacy and heart failure: a systematic review. J Cardiovasc Nurs. 2016;31:121-130. 10.1097/JCN.0000000000000229 PMC4577469 25569150

[59] Nahum-Shani I , Dziak JJ, Walton MA, Dempsey W. Hybrid experimental designs for intervention development: what, why, and how. Adv Methods Pract Psychol Sci. 2022;5:25152459221114279. 10.1177/25152459221114279 PMC10024531 36935844

