# Supplementary material for: Early psychosocial-behavioral phenotypes associated with subsequent engagement and longitudinal outcomes in a digital health intervention for heart failure
Source: Ann Behav Med. 2026 Nov 2;60(1):kaag066. doi: 10.1093/abm/kaag066 (PMC13725594; doi:10.1093/abm/kaag066)
Supplement: Supplementary file 1 [file kaag066_supplementary_data.docx]

**Supplementary Table S1.** Study Variables, Data Sources, Scoring, and Analytic Purpose

| **Variable** | **Measure** | **Data Source** | **Scoring** | **Analysis Use** |
| --- | --- | --- | --- | --- |
| ***Baseline Participant Characteristics*** | | | | |
| Age | Self-report | Baseline survey | Years (continuous) | Cluster Profiling, Covariate |
| Sex | Self-report | Baseline survey | Male / Female | Cluster Profiling, Covariate |
| Race/ethnicity | Self-report | Baseline survey | NH White / Minority | Cluster Profiling, Covariate |
| Prior tracker ownership | Self-report | Baseline survey | Yes / No | Cluster Profiling, Covariate |
| Study group | Randomization | Study assignment | Control / Intervention | Cluster Profiling, Covariate |
| HF duration | Self-report | Baseline survey | ≤1 yr / 2–4 yr / ≥5 yr | Cluster Profiling |
| Employment status | Self-report | Baseline survey | Employed / Retired / Disabled / Unemployed | Cluster Profiling |
| Smartphone type | Self-report | Baseline survey | Android / iOS | Cluster Profiling |
| Food insecurity | Upstream Risk Screening Tool | Baseline survey | Yes / No | Cluster Profiling |
| Housing concern | Upstream Risk Screening Tool | Baseline survey | Yes / No | Cluster Profiling |
| Transportation difficulty | Upstream Risk Screening Tool | Baseline survey | Yes / No | Cluster Profiling |
| Perceived neighborhood safety | Upstream Risk Screening Tool | Baseline survey | Yes / No | Cluster Profiling |
| ***Variables Used for Cluster Analysis*** | | | | |
| Momentary anxiety | Single-item VAS | EMA (mean of AM Week 1) | 0–100 (0 = calm; 100 = anxious) | Clustering input |
| Momentary depressed mood | Single-item VAS | EMA (mean of AM Week 1) | 0–100 (0 = happy; 100 = depressed) | Clustering input |
| Physical activity motivation | Single-item VAS | EMA (mean of AM Week 1) | 0–100 (0 = none; 100 = high) | Clustering input |
| Self-efficacy for HF self-care | SCHFI-6 (α = .83) | SCHFI-6 | 0–100 (higher = better) | Clustering input |
| Social connectedness | 4-item composite: (phone, in-person, faith-based, group interactions) | Sociodemographic questionnaire | Composite index: 0–8 (higher = more connected) | Clustering input |
| Education | Single item | Sociodemographic questionnaire | 0 = ≤HS; 1 = >HS | Clustering input |
| Financial hardship | Single item | Sociodemographic questionnaire | 0 = No; 1 = Yes | Clustering input |
| Partnered status | Single item | Sociodemographic questionnaire | 0 = No; 1 = Yes | Clustering input |
| Smart scale engagement | Proportion of days synced | Device logs (days 1–28) | 0-1 (proportion of days) | Clustering input |
| Activity tracker engagement | Proportion of days synced | Device logs (days 1–28) | 0-1 (proportion of days) | Clustering input |
| ***Momentary Psychological States (Longitudinal)*** | | | | |
| Momentary anxiety | Single-item VAS | EMA (Wks 1, 7, 13, 23; 7-day AM means) | 0–100 (0 = calm; 100 = anxious) | LMM Outcome |
| Momentary depressed mood | Single-item VAS | EMA (Wks 1, 7, 13, 23; 7-day AM means) | 0–100 (0 = happy; 100 = depressed) | LMM Outcome |
| Physical activity motivation | Single-item VAS | EMA (Wks 1, 7, 13, 23; 7-day AM means) | 0–100 (0 = none; 100 = high) | LMM Outcome |
| ***Patient-Reported Health Outcomes*** | | | | |
| Functional status | KCCQ-FS (α = .93) | Survey (BL, Wks 6, 12, 24) | 0–100 (higher = better) | LMM Outcome |
| Quality of life | KCCQ-QoL (α = .78) | Survey (BL, Wks 6, 12, 24) | 0–100 (higher = better) | LMM Outcome |
| Self-care behaviors | EHFScBS-9 (α = .80) | Survey (BL, Wks 6, 12, 24) | 0–100 (higher = better) | LMM Outcome |
| HF knowledge | AHFKT (α = .87) | Survey (BL, Wks 6, 12, 24) | 0–100 (higher = better) | LMM Outcome |
| ***Device Engagement Outcome (weeks 5-24)*** | | | | |
| Smart scale engagement | Days synced | Device logs (weeks 5-24) | Proportion of days (0-1) | Kruskal-Wallis outcome |
| Activity tracker engagement | Days synced | Device logs (weeks 5-24) | Proportion of days (0-1) | Kruskal-Wallis outcome |

***Note.*** VAS = visual analog scale; EMA = ecological momentary assessment; SCHFI-6 = Self-Care of Heart Failure Index, Confidence subscale; KCCQ-FS = Kansas City Cardiomyopathy Questionnaire, Functional Status; KCCQ-QoL = KCCQ Quality of Life subscale; EHFScBS-9 = European Heart Failure Self-Care Behavior Scale; AHFKT = Atlanta Heart Failure Knowledge Test; LMM = linear mixed-effects model.

**Supplementary Table S2.** EMA Missingness by Phenotype and Study Wave (N = 175)

|  | **Week 1** | **Week 7** | **Week 13** | **Week 23** |
| --- | --- | --- | --- | --- |
| ***Daily Response Missingness (%)*** | | | | |
| Challenged (n = 46) | 21.4 | 35.4 | 46.6 | 49.7 |
| Activated (n = 55) | 19.5 | 40.3 | 47.5 | 48.3 |
| Engaged (n = 74) | 13.3 | 20.5 | 22.6 | 29.9 |
| ***Fully Missing Bursts, n (%)*** | | | | |
| Challenged (n = 46) | 0 (0.0) | 6 (13.0) | 10 (21.7) | 16 (34.8) |
| Activated (n = 55) | 0 (0.0) | 9 (16.4) | 17 (30.9) | 17 (30.9) |
| Engaged (n = 74) | 0 (0.0) | 3 (4.1) | 6 (8.1) | 13 (17.6) |
| ***Contributing Days per Burst, M (SD)*** | | | | |
| Challenged (n = 46) | 5.5 (1.6) | 5.2 (1.9) | 4.8 (2.2) | 5.4 (2.0) |
| Activated (n = 55) | 5.6 (1.8) | 5.0 (1.8) | 5.3 (2.0) | 5.2 (2.0) |
| Engaged (n = 74) | 6.1 (1.6) | 5.8 (1.6) | 5.9 (1.5) | 6.0 (1.5) |

***Note.*** Daily response missingness reflects the percentage of scheduled morning EMA responses that were missing prior to aggregation. Fully missing bursts indicate participants with zero responses during a 7-day assessment wave. Contributing days per burst were calculated among participants with ≥1 response.

**Supplementary Table S3.** Patient-Reported Outcome Missingness by Phenotype and Timepoint

| **Timepoint** | **Phenotype** | **Functional Status**  **n (%)** | **Quality of Life**  **n (%)** | **HF Knowledge**  **n (%)** | **Self-Care**  **n (%)** |
| --- | --- | --- | --- | --- | --- |
| **Baseline** | Challenged (n = 46) | 0 (0) | 0 (0) | 0 (0) | 0 (0) |
|  | Activated (n = 55) | 0 (0) | 0 (0) | 0 (0) | 0 (0) |
|  | Engaged (n = 74) | 0 (0) | 0 (0) | 0 (0) | 0 (0) |
| **Week 6** | Challenged | 4 (8.7) | 4 (8.7) | 6 (13.0) | 6 (13.0) |
|  | Activated | 6 (10.9) | 6 (10.9) | 6 (10.9) | 6 (10.9) |
|  | Engaged | 2 (2.7) | 2 (2.7) | 2 (2.7) | 2 (2.7) |
| **Week 12** | Challenged | 8 (17.4) | 8 (17.4) | 9 (19.6) | 9 (19.6) |
|  | Activated | 8 (14.5) | 8 (14.5) | 10 (18.2) | 12 (21.8) |
|  | Engaged | 3 (4.1) | 3 (4.1) | 5 (6.8) | 6 (8.1) |
| **Week 24** | Challenged | 10 (21.7) | 10 (21.7) | 13 (28.3) | 13 (28.3) |
|  | Activated | 14 (25.5) | 14 (25.5) | 14 (25.5) | 14 (25.5) |
|  | Engaged | 8 (10.8) | 8 (10.8) | 9 (12.2) | 9 (12.2) |

**Supplementary Figure S1.** Cluster selection diagnostics for k-medoids clustering.

***Note.*** (A) Average silhouette width across k = 2–10 clusters. (B) Total within-cluster dissimilarity (elbow plot). (C) Bootstrap stability (500 resamples) of the three-cluster solution; dashed line indicates the 0.75 Jaccard index threshold for stable clusters. Red dots in panels A and B indicate the selected k = 3 solution.

**Supplementary Table S4.** Clustering Variable Descriptives by Psychosocial–Behavioral Phenotype (N = 175)

| **Variable** | **Challenged Survivors (n = 46)** | **Activated Learners**  **(n = 55)** | **Engaged Self-Regulators**  **(n = 74)** |
| --- | --- | --- | --- |
| ***Psychological*** | | | |
| Anxiety (0–100), M (SD) | 37.9 (23.5) | 44.7 (20.7) | 25.3 (18.6) |
| Depressed mood (0–100), M (SD) | 40.2 (21.9) | 41.5 (18.0) | 24.0 (16.9) |
| PA motivation (0–100), M (SD) | 52.1 (21.6) | 55.6 (21.3) | 67.6 (21.9) |
| Self-efficacy (0–100), M (SD) | 56.1 (20.0) | 49.6 (20.9) | 56.5 (21.6) |
| ***Social*** | | | |
| Social connectedness (0–8), M (SD) | 3.9 (1.7) | 4.2 (2.1) | 4.8 (1.9) |
| College education, n (%) | 0 (0) | 55 (100) | 66 (89) |
| Financial hardship (yes), n (%) | 35 (76) | 52 (95) | 7 (10) |
| Partnered (yes), n (%) | 8 (17) | 12 (22) | 68 (92) |
| ***Digital Engagement*** | | | |
| Weeks 1-4 (28 days), % of days, M (SD) | | | |
| Tracker use | 79 (32) | 87 (23) | 83 (30) |
| Scale use | 51 (32) | 66 (28) | 67 (31) |
| Weeks 5–24 (140 days), % of days, M (SD) | | | |
| Tracker use | 59 (37) | 67 (35) | 77 (31) |
| Scale use | 33 (31) | 43 (31) | 59 (32) |
| Full study period (168 days), % of days, M (SD) | | | |
| Tracker use | 62 (35) | 71 (30) | 78 (29) |
| Scale use | 36 (30) | 47 (28) | 61 (31) |

***Note.*** Digital engagement reflects the percentage of days each device was synced within each observation window.

**Supplementary Table S5.** Pairwise Comparisons of Baseline Characteristics and Subsequent Device Engagement (Weeks 5–24) Across Phenotypes

| **Variable** | **Comparison** | ***Z*** | ***P* (unadj.)** | ***P* (BH adj.)** | **Interpretation** |
| --- | --- | --- | --- | --- | --- |
| ***Age (years), H = 21.15, P < .001*** | | | | | |
|  | Activated vs. Challenged | 1.21 | .227 | .227 | NS |
|  | Activated vs. Engaged | −3.20 | .001 | .002 | E > A |
|  | Challenged vs. Engaged | −4.32 | <.001 | <.001 | E > Ch |
| ***Functional status (KCCQ), H = 48.37, P < .001*** | | | | | |
|  | Activated vs. Challenged | 0.01 | .993 | .993 | NS |
|  | Activated vs. Engaged | −5.97 | <.001 | <.001 | E > A |
|  | Challenged vs. Engaged | −5.67 | <.001 | <.001 | E > Ch |
| ***Quality of life (KCCQ), H = 42.01, P < .001*** | | | | | |
|  | Activated vs. Challenged | 0.23 | .816 | .816 | NS |
|  | Activated vs. Engaged | −5.45 | <.001 | <.001 | E > A |
|  | Challenged vs. Engaged | −5.41 | <.001 | <.001 | E > Ch |
| ***HF knowledge (AHFKT), H = 15.47, P < .001*** | | | | | |
|  | Activated vs. Challenged | 2.80 | .005 | .008 | A > Ch |
|  | Activated vs. Engaged | −0.94 | .348 | .348 | NS |
|  | Challenged vs. Engaged | −3.87 | <.001 | <.001 | E > Ch |
| ***Self-care (EHFScBS), H = 0.39, P = .823*** | | | | | |
|  | No post hoc tests conducted | — | — | — | — |
| ***Tracker use, weeks 5–24, H = 7.05, P = .03*** | | | | | |
|  | Activated vs. Challenged | 1.12 | .261 | .261 | NS |
|  | Activated vs. Engaged | −1.49 | .135 | .203 | NS |
|  | Challenged vs. Engaged | −2.61 | .009 | .027 | E > Ch |
| ***Scale use, weeks 5–24, H = 18.43, P < .001*** | | | | | |
|  | Activated vs. Challenged | 1.58 | .114 | .114 | NS |
|  | Activated vs. Engaged | −2.62 | .009 | .013 | E > A |
|  | Challenged vs. Engaged | −4.17 | <.001 | <.001 | E > Ch |

***Note.*** Pairwise comparisons from Dunn’s tests following significant Kruskal–Wallis omnibus tests. p (BH adj.) = Benjamini–Hochberg adjusted p-value. A = Activated; Ch = Challenged; E = Engaged; NS = not significant.

**Supplementary Table S6.** Descriptive EMA Levels by Phenotype and Study Wave (N = 175)

| **Study Wave** | **n** | **Anxiety, M (SD)** | **Depressed Mood, M (SD)** | **PA Motivation, M (SD)** |
| --- | --- | --- | --- | --- |
| ***Challenged (n = 46)*** | | | | |
| Week 1 | 46 | 37.9 (23.5) | 40.2 (21.9) | 52.1 (21.6) |
| Week 7 | 40 | 37.9 (26.6) | 39.4 (26.6) | 50.7 (26.8) |
| Week 13 | 36 | 37.8 (25.9) | 40.9 (26.4) | 48.9 (23.3) |
| Week 23 | 30 | 37.4 (25.5) | 37.8 (24.4) | 51.0 (25.9) |
| ***Activated (n = 55)*** | | | | |
| Week 1 | 55 | 44.7 (20.7) | 41.5 (18.0) | 55.6 (21.3) |
| Week 7 | 46 | 42.1 (24.9) | 40.2 (25.4) | 56.3 (25.0) |
| Week 13 | 38 | 44.8 (28.0) | 41.4 (24.9) | 53.1 (25.5) |
| Week 23 | 38 | 37.1 (22.9) | 36.9 (22.3) | 56.0 (25.7) |
| ***Engaged (n = 74)*** | | | | |
| Week 1 | 74 | 25.3 (18.6) | 24.0 (16.9) | 67.6 (21.9) |
| Week 7 | 71 | 24.8 (19.9) | 23.5 (18.3) | 66.5 (23.9) |
| Week 13 | 68 | 25.8 (21.1) | 24.6 (20.5) | 65.9 (24.9) |
| Week 23 | 61 | 24.5 (22.2) | 25.6 (22.1) | 65.7 (24.8) |

**Note.** Values represent observed means and standard deviations of person-level burst means (averaged across 7 consecutive days of morning ecological momentary assessment).

**Supplementary Table S7.** Linear Mixed-Effects Model Full Results for Momentary Psychological States by Phenotype Across Study Waves (N = 175)

|  | **Anxiety** | **Depressed Mood** | **PA Motivation** |
| --- | --- | --- | --- |
| ***Random Effects*** | | | |
| ICC | .69 | .71 | .77 |
| σ² participant (SD) | 352.8 (18.8) | 332.0 (18.2) | 432.3 (20.8) |
| σ² residual (SD) | 160.6 (12.7) | 136.7 (11.7) | 128.9 (11.4) |
| ***Type III Fixed Effects*** | | | |
| Phenotype | 13.57 **(<.001)** | 14.58 **(<.001)** | 8.90 **(<.001)** |
| Study wave | 1.48 (.218) | 1.14 (.332) | 1.55 (.201) |
| Phenotype × wave | 1.17 (.322) | 0.96 (.451) | 0.31 (.932) |
| ***Pairwise Contrasts: Week 1, b (P)*** | | | |
| Engaged – Challenged | −12.63 (.009) | −16.28 (<.001) | 15.51 (.002) |
| Engaged – Activated | −19.48 (<.001) | −17.50 (<.001) | 11.95 (.014) |
| Challenged – Activated | −6.86 (.286) | −1.23 (.957) | −3.56 (.733) |
| ***Pairwise Contrasts: Week 7, b (P)*** | | | |
| Engaged – Challenged | −14.71 (.002) | −17.56 (<.001) | 17.17 (<.001) |
| Engaged – Activated | −17.68 (<.001) | −17.22 (<.001) | 10.63 (.039) |
| Challenged – Activated | −2.98 (.803) | 0.34 (.997) | −6.54 (.375) |
| ***Pairwise Contrasts: Week 13, b (P)*** | | | |
| Engaged – Challenged | −14.13 (.005) | −17.74 (<.001) | 17.03 (<.001) |
| Engaged – Activated | −20.24 (<.001) | −18.21 (<.001) | 13.27 (.008) |
| Challenged – Activated | −6.11 (.424) | −0.48 (.994) | −3.76 (.734) |
| ***Pairwise Contrasts: Week 23, b (P)*** | | | |
| Engaged – Challenged | −14.11 (.007) | −11.56 (.024) | 14.26 (.008) |
| Engaged – Activated | −12.47 (.012) | −12.13 (.010) | 11.28 (.032) |
| Challenged – Activated | 1.64 (.942) | −0.58 (.992) | −2.98 (.829) |

**Supplementary Table S8.** Sensitivity Analysis: Primary vs. Complete-Case EMA Models

|  | **Primary (N = 175)** | **Complete-Case (n = 121)** |
| --- | --- | --- |
| ***Anxiety*** | | |
| Phenotype, F (*P*) | 13.57 (<.001) | 7.51 (<.001) |
| Study wave, F (*P*) | 1.48 (.218) | 1.32 (.268) |
| Phenotype × wave, F (*P*) | 1.17 (.322) | 1.15 (.331) |
| ***Depressed Mood*** | | |
| Phenotype, F (*P*) | 14.58 (<.001) | 8.78 (<.001) |
| Study wave, F (*P*) | 1.14 (.332) | 2.05 (.107) |
| Phenotype × wave, F (*P*) | 0.96 (.451) | 1.27 (.273) |
| ***Physical Activity Motivation*** | | |
| Phenotype, F (*P*) | 8.90 (<.001) | 5.61 (.005) |
| Study wave, F (*P*) | 1.55 (.201) | 1.47 (.223) |
| Phenotype × wave, F (*P*) | 0.31 (.932) | 0.37 (.898) |

**Supplementary Table S9.** Table of Model Comparisons

| **Outcome** | **Model** | **AIC** | **BIC** | **Δχ²** | **Δdf** | ***P*** |
| --- | --- | --- | --- | --- | --- | --- |
| Functional Status | Step 1: Unconditional Growth | 5,218.2 | 5,236.1 | — | — | — |
|  | Step 2: + Phenotype | 5,173.7 | 5,200.5 | 48.49 | 2 | < .001 |
|  | **Step 3: + Covariates** | **5,167.4** | **5,216.4** | **16.37** | **5** | **0.006** |
|  | Step 4: + Phenotype × Time | 5,165.8 | 5,223.7 | 5.61 | 2 | 0.060 |
| Quality of Life | Step 1: Unconditional Growth | 5,514.4 | 5,541.2 | — | — | — |
|  | Step 2: + Phenotype | 5,472.4 | 5,508.1 | 46.04 | 2 | < .001 |
|  | **Step 3: + Covariates** | **5,472.7** | **5,530.6** | **9.74** | **5** | **0.083** |
|  | Step 4: + Phenotype × Time | 5,475.0 | 5,541.8 | 1.68 | 2 | 0.431 |
| HF Knowledge | Step 1: Unconditional Growth | 4,133.1 | 4,159.8 | — | — | — |
|  | Step 2: + Phenotype | 4,122.4 | 4,157.9 | 14.73 | 2 | < .001 |
|  | Step 3: + Covariates | 4,115.2 | 4,172.9 | 17.20 | 5 | 0.004 |
|  | **Step 4: + Phenotype × Time** | **4,108.8** | **4,175.4** | **10.41** | **2** | **0.005** |
| Self-Care | Step 1: Unconditional Growth | 5,036.7 | 5,054.4 | — | — | — |
|  | Step 2: + Phenotype | 5,038.3 | 5,064.9 | 2.33 | 2 | 0.312 |
|  | **Step 3: + Covariates** | **5,036.8** | **5,085.6** | **11.49** | **5** | **0.042** |
|  | Step 4: + Phenotype × Time | 5,040.2 | 5,097.8 | 0.64 | 2 | 0.726 |

**Supplementary Table S10.** Pattern-Mixture Model Sensitivity Analyses for HF Health Outcomes (N = 175)

|  | **Functional Status** | **Quality of Life** | **HF Knowledge** | **Self-Care** | **Robust?** |
| --- | --- | --- | --- | --- | --- |
| ***Model 1: Overall Dropout Effects*** | | | | | |
| Dropout (main effect) | .292 | .254 | .749 | .301 | Yes |
| Time × Dropout | .772 | .571 | .577 | .622 | Yes |
| Phenotype (Challenged) | < .001 | < .001 | .025 | .331 | Yes |
| Phenotype (Activated) | < .001 | < .001 | .855 | .799 | Yes |
| Time × Activated | — | — | .003 | — | Yes |
| ***Model 2: Phenotype × Dropout Effects*** | | | | | |
| Challenged × Dropout | .269 | .583 | .966 | .807 | Yes |
| Activated × Dropout | .323 | .205 | .919 | .091 | Yes |
| Phenotype (Challenged) | < .001 | < .001 | .040 | .491 | Yes |
| Phenotype (Activated) | < .001 | < .001 | .834 | .278 | Yes |
| Time × Activated | — | — | .004 | — | Yes |
| ***Model 3: Three-Way Interaction (HF Knowledge only)*** | | | | | |
| Time × Activated | — | — | .012 | — | Yes |
| Time × Ch × Dropout | — | — | .498 | — | Yes |
| Time × A × Dropout | — | — | .068 | — | Yes |

***Note.*** Values are *p*-values from pattern-mixture models. Model 1 added a Week 24 dropout indicator and time × dropout interaction to the primary model. Model 2 additionally included phenotype × dropout interactions. Model 3 (HF Knowledge only) included the full three-way phenotype × time × dropout interaction. Dashes indicate terms not applicable to that outcome. Ch = Challenged; A = Activated.

**Supplementary Table S11.** Tipping Point Sensitivity Analyses for Primary Health Outcome Findings (*N* = 175)

| **Effect Tested** | **Tipping Point (δ)** | ***P* at δ = 0** | ***P* at δ = −10** |
| --- | --- | --- | --- |
| ***Time Effects*** | | | |
| Functional Status | −8.5 | < .001 | .105 |
| Quality of Life | Not reached | < .001 | < .001 |
| HF Knowledge^a^ | Not reached | < .001 | < .001 |
| Self-Care | Not reached | < .001 | < .001 |
| ***Phenotype Effects*** | | | |
| FS: Challenged vs Engaged | Not reached | < .001 | < .001 |
| FS: Activated vs Engaged | Not reached | < .001 | < .001 |
| QoL: Challenged vs Engaged | Not reached | < .001 | < .001 |
| QoL: Activated vs Engaged | Not reached | < .001 | < .001 |
| ***HF Knowledge Interaction*** | | | |
| Time × Activated (δ < 0)^a^ | Not reached | .003 | < .001 |
| Time × Activated (δ > 0)^a,b^ | +7.5 | .003 | — |

^a^ No established MCID for HF Knowledge (AHFKT); 2.8 points (½ SD) used as benchmark.

ᵇ Positive δ (0 to +10) represents the clinically implausible scenario in which dropouts improved more than MAR-predicted values.

**Supplementary Text S1: Missing Data and Sensitivity Analyses**

**Missing Data**

EMA Data. EMA response rates declined over the study period across all phenotypes. After aggregating daily responses to person-wave means, 14% of person-wave observations (97 of 700) had no EMA data for that wave, occurring only at follow-up waves (Weeks 7, 13, and 23) and most frequently in the Challenged phenotype (35% missing by Week 23, compared to 31% for Activated and 18% for Engaged). All participants contributed EMA data at Week 1; 162 (93%) contributed data at one or more additional waves. Among participants with data for a given wave, burst-level means were based on an average of 4.8–6.1 contributing days (SD = 1.5–2.2), supporting the stability of aggregated estimates. LMMs accommodate missing person-wave observations under the missing at random (MAR) assumption; complete-case sensitivity analyses restricted to participants with data at all four waves (n = 121) supported the robustness of findings to differential missingness. Detailed EMA missingness by phenotype and study wave is reported in Supplementary Table S2.

For patient-reported outcomes, missingness followed predominantly monotone dropout patterns and was similarly differential by phenotype: by Week 24, missingness reached 22–28% in the Challenged and 26% in the Activated groups, compared to 11–12% in the Engaged group. Logistic regression indicated that younger age and intervention group assignment predicted Week 24 missingness; both variables are included as covariates in the primary models.

**Sensitivity Analyses for Potential MNAR Departures**

To evaluate robustness to potential departures from the MAR assumption for health outcome models, we conducted pattern-mixture models and tipping point analyses.

Pattern-Mixture Models. Pattern-mixture models incorporated a binary Week 24 dropout indicator as a fixed effect. In initial models, neither dropout main effects nor time × dropout interactions reached significance for any outcome (all ps > .05), indicating that completers and dropouts did not differ meaningfully in baseline levels or rates of change. When phenotype × dropout interactions were added to address differential attrition across phenotypes, no interactions reached significance (all ps > .05), and all primary findings remained stable, including the HF knowledge phenotype × time interaction (Activated: p = .004). For HF knowledge specifically, a three-way pattern-mixture model (phenotype × time × dropout) confirmed that the Activated phenotype's flat trajectory was not an artifact of differential attrition (time × Activated: b = −0.15, p = .012; three-way interactions: p = .50 and p = .07).

Tipping Point Analyses. Tipping point analyses applied increasingly pessimistic delta adjustments (δ = 0 to −10) to post-dropout observations, representing hypothetical scenarios in which dropouts fared worse than MAR-predicted values. For the KCCQ outcomes, this range represents departures up to twice the established MCID of 5 points. For self-care, this exceeds the estimated minimal important change of approximately 8 points. For HF knowledge, no established MCID exists.

Time effects for quality of life, HF knowledge, and self-care remained significant across all δ values tested (all ps < .001 at δ = −10). The functional status time effect was the most sensitive, tipping at δ = −8.5 (1.7 times the MCID of 5 points). Phenotype effects on functional status and quality of life showed no tipping point; estimates strengthened under pessimistic assumptions due to disproportionate dropout in the Challenged and Activated groups. The HF knowledge phenotype × time interaction remained significant across all negative δ values and tipped only in the positive direction (δ = +7.5), a scenario requiring dropouts to have improved substantially beyond MAR-predicted values. Collectively, these analyses indicate that the primary findings are robust to plausible MNAR departures.
